# Evaluation of the Impact of *Alternanthera philoxeroides* (Mart.) Griseb. Extract on Memory Impairment in D-Galactose-Induced Brain Aging in Mice through Its Effects on Antioxidant Enzymes, Neuroinflammation, and Telomere Shortening

**DOI:** 10.3390/molecules29020503

**Published:** 2024-01-19

**Authors:** Possatorn Aon-im, Orawan Monthakantirat, Supawadee Daodee, Yaowared Chulikhit, Nattapatsorn Sriya, Chantana Boonyarat, Thanut Chumwangwapee, Charinya Khamphukdee, Anake Kijjoa

**Affiliations:** 1 Graduate School of Pharmaceutical Sciences, Khon Kaen University, Khon Kaen 40002, Thailand; possa.torn@kkumail.com; 2 Faculty of Pharmaceutical Sciences, Khon Kaen University, Khon Kaen 40002, Thailand; oramon@kku.ac.th (O.M.); csupawad@kku.ac.th (S.D.); yaosum@kku.ac.th (Y.C.); nattapatsorn_sriya@kkumail.com (N.S.); chaboo@kku.ac.th (C.B.); thacho@kku.ac.th (T.C.); 3 ICBAS-Instituo de Ciências Biomédicas Abel Salazar and CIIMAR, Universidade do Porto, Rua de Jorge Viterbo Ferreira 228, 4050-313 Porto, Portugal

**Keywords:** *Alternanthera philoxeroides*, brain aging, memory deficits, D-galactose-induced aging, neuroinflammation, telomere shortening

## Abstract

Aging is a well-known factor that accelerates brain deterioration, resulting in impaired learning and memory functions. This current study evaluated the potential of an extract of *Alternanthera philoxeroides* (AP), an edible flavonoid-rich plant, to ameliorate D-galactose-induced brain aging in male mice. Chronic administration of D-galactose (150 mg/kg/day) in mice mimicked the characteristics of aging by accelerating senescence via downregulation of the following telomere-regulating factors: mouse telomerase reverse transcriptase (mTERT) and mouse telomeric repeat-binding factors 1 (mTRF1) and 2 (mTRF2). D-galactose also decreased the activities of the antioxidant enzymes catalase (CAT) and superoxide dismutase (SOD), while increasing expression of neuroinflammatory cytokines in the frontal cortex and hippocampus. Daily treatment of D-galactose-induced aging mice with AP at 250 and 500 mg/kg/day or vitamin E (100 mg/kg/day) significantly increased the activities of SOD and CAT, as well as expression of mTERT, mTRF1, and mTRF2, which are involved in telomere stabilization, but decreased the levels of proinflammatory cytokines IL-1β, IL-6, and TNF-α. In the behavioral portion of the study, AP improved aging-related cognitive deficits in short-term memory as shown by the Y-maze task and the novel object recognition test (NORT) and long-term memory as shown by the Morris water maze test (MWMT). The flavones kaempferol-*O*-glucoside (**1**), quercetin (**2**), alternanthin B (**3**), demethyltorosaflavone D (**4**), and chrysoeriol-7-*O*-rhamnoside (**5**), which could be responsible for the observed effects of AP in the D-galactose-induced aging mice, were identified by HPLC analysis.

## 1. Introduction

According to the World Health Organization (WHO), more than 55 million people currently have dementia worldwide, over 60% of whom live in low- and middle-income countries, and there are nearly 10 million new cases every year [1]. Dementia is caused by many different diseases or injuries that directly and indirectly damage the brain, with the prevalence increasing with age from approximately 1% for people aged in their 60s up to 5% for people in their 70s and 15% for those in their 80s [1,2]. Therefore, it is important to understand how age impacts cognition and what preventative strategies could preserve cognition into advanced age [3]. Aging is also known as senescence and is a natural and inevitable process that occurs in most organisms due to a gradual deterioration of biological functions and physiological processes over time [4]. Aging also directly affects cognitive functions, such as the regulation of language, memory, attention, movement, and some executive functions such as problem solving, judgment, and planning [5]. 

Reactive oxygen species (ROS), resulting from biological processes such as respiration, can cause damage to biomolecules that accumulate and contribute to aging via oxidative stress [6]. ROS are widely recognized to cause brain deterioration in the elderly and impair memory and learning functions [6]. Since the brain is a lipid-rich organ, with low activity of the antioxidant defense system and high oxygen consumption, it is sensitive to oxidative stress due to excessive oxidant production [7]. For these reasons, the brain is more vulnerable to oxidative stress than other organs. 

Although there are endogenous lipid-soluble antioxidant enzymes, namely superoxide dismutase (SOD), catalase (CAT), and glutathione peroxidase (GPx), that destroy ROS in the brain [8], this system becomes weakened with age and fails to cope with excessive ROS production, resulting in damage to the cellular composition. Since the damaged cells cannot perform regular functions, they become senescent. Neurons that undergo oxidative damage in advanced age are not repaired or regenerated, causing neurodegeneration and age-related memory deficits [9].

Chronic neuroinflammatory processes in which microglia are hyperactivated and overproduce proinflammatory cytokines such as IL-1ß, IL-6, and TNF-α are a central feature of neurodegeneration [9,10,11]. IL-1ß is an important initiator of the immune response that plays a key role in the cellular inflammatory cascade and neuronal degeneration. IL-1ß induces IL-6 production and stimulates inducible nitric oxide synthase (iNOS) activity, which triggers oxidative stress [12]. It also enhances neuronal acetylcholinesterase activity, resulting in the production of choline neurotransmitters. IL-6 and TNF-α play important roles in regulating the cytokine cascade during an inflammatory response [13]. These neuroinflammatory cytokines also respond to the inflammatory process in eradicating necrotic cells and damaged tissues. Chronic inflammation, which is known as inflammageing, represents an exclusively pathological effect of the aging process [11,13,14].

Another factor that contributes to aging is telomere attrition, which is a gradual shortening of telomeres with each cell division. Telomeres shorten due to insufficient telomerase expression [15]. Telomerase production is regulated by telomerase reverse transcriptase (TERT), a catalytic subunit that plays a major role in telomere elongation. Telomerase activity is regulated by the shelterin complex that consists of telomeric repeat-binding factors 1 and 2 (TRF1 and TRF2). These components are deprived with age, and telomere protection is attenuated [16,17]. Telomeres play a crucial role in maintaining genomic stability and integrity during cell replication [16]. When telomeres become critically short, a cell will enter a state of cell cycle arrest and becomes senescent. Tissue degeneration can develop if accumulated senescent cells are not eliminated. Additionally, cells responsible for tissue regeneration are not able to function properly [18]. As microglia are an assembly of proliferative cells, it is likely that their continuous self-renewal and the subsequent telomere shortening affect their function. Indeed, the shortening of microglia telomeres has been reported to be involved in aging and memory loss [19]. 

Since the multi-pathogenesis of dementia is involved in aging, the classical approach that modulates only one target may not be suitable to tackle this disease. Therefore, searching for multi-target modulations, based on the pathologic cascade, has been an effective strategy to delay the onset of cognitive symptoms in age-related diseases [20]. 

D-galactose (D-Gal) is well documented as an activator of oxidative stress and inflammation [21]. Chronic administration of D-Gal (150–300 mg/kg/day) leads to oxidative stress, inflammatory response, and cellular apoptosis, especially in the hippocampus, which directly regulates memory and learning functions [22]. These phenomena are similar to the aging process and acceleration of the senescence model, demonstrating the mechanism of neurological impairment. Therefore, D-Gal-induced neurotoxicity in mice is a useful model for studying neurodegeneration mechanisms and for screening neuroprotective herbal extracts or compounds [23]. 

*Alternanthera philoxeroides* (Mart.) Griseb. (family Amaranthaceae) is an edible herb which is used in Thai folk medicine to ameliorate blood conditions and to treat post-natal depression [24]. Previous chemical studies of AP revealed the presence of cytotoxic pentacyclic triterpene saponins (philoxeroidesides A–D) and antiviral pentacyclic triterpenes (chikusetsusaponin Iva and calenduloside E) [25]. We have previously described the isolation of the *C*-glycosylated flavones alternanthin and alternanthin B, the flavone glycoside demethyltorosaflavone B, and the *E*-propenoic acid substituted flavone torosaflavone E, as well as demethyltorosaflavone D, luteolin 8-C-*E*-propenoic acid, and chrysoeriol 7-*O*-rhamnoside from the ethanol extract of AP collected in Khon Kaen, Thailand [26].

In vivo studies of the ethanol extract of *A. philoxeroides* revealed a capacity to improve depression and dementia symptoms in ovariectomized (OVX) mice. These results led us to hypothesize that these effects were due to the presence of flavonoids, which may act as phytoestrogens via estrogen-receptor-mediated reinforcement of a conserved cAMP response element-binding protein (*CREB*) and abnormal brain-derived neurotrophic factor (*BDNF*) genes’ transcription in the hippocampus and frontal cortex to mediate neurogenesis and neuroplasticity processes [26].

The AP extract also lowered the levels of pro-inflammatory cytokines (IL-1ß, IL-6, and TNF-α) in mouse brains [27]. Finally, alternanthin and alternanthin B, demethyltorosaflavone B, torosaflavone E, demethyltorosaflavone D, luteolin 8-C-*E*-propenoic acid, and chrysoeriol 7-*O*-rhamnoside were all found to inhibit the activities of monoamine oxidase enzymes [26] and reduce β-amyloid aggregation [27].

The presence of flavonoids in the AP extract led us to hypothesize that it could reduce the incidence of cognitive dysfunction by the attenuation of oxidative stress and neuroinflammatory responses caused by aging. Therefore, the aim of this study was to determine if the AP extract had antidementia effects on D-Gal-induced aging in mice by alleviating oxidative stress, neuroinflammation, and telomere shortening. 

## 2. Results

### 2.1. Preparation of the Ethanol Extract of A. philoxeroides (AP)

Whole plants of *A. philoxeroides* were collected from Khon Kaen province, Thailand, in April 2021. A voucher specimen, KKPH010102986, was deposited at the herbarium of the Faculty of Pharmaceutical Sciences, Khon Kaen University, Thailand. Dried power (1 kg) of *A. philoxeroides* was refluxed with EtOH (3 × 1 L) at 70 °C for 2 h and filtered. The solution was concentrated under reduced pressure at 45–50 °C to give 108.8 g of crude extract, which was subsequently freeze-dried. The crude extract (AP) was kept at −20 °C during the experiment.

### 2.2. Determination of Total Phenolic and Flavonoid Contents and Radical Scavenging Capacity of AP 

The total phenolic and total flavonoid contents of AP were determined from their calibration curves (r^2^ = 0.9991 and r^2^ = 0.9991, respectively). The total phenolic content was 43.50 mg of gallic acid equivalent (GAE)/g extract and the total flavonoid content was 57.34 mg of quercetin equivalent (QE)/g extract. The antioxidant activity of AP was determined by oxygen radical absorbing capacity (ORAC) and ferric reducing antioxidant power (FRAP) assays. The ORAC value of AP was 563 µM Trolox equivalent (TE)/g extract, while the FRAP value of AP was 151.36 µM Trolox equivalent antioxidant capacity (TEAC)/g extract. 

### 2.3. Phytochemical Analysis of AP by High-Performance Liquid Chromatography (HPLC) and the Validation Method

**Figure 1:**
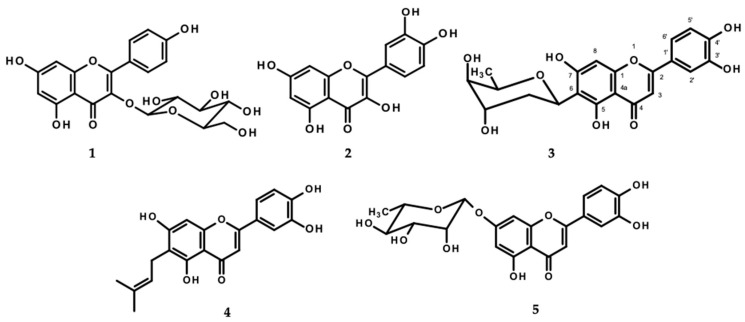
Structures of kaempferol-*O*-glucoside (**1**), quercetin (**2**), alternanthin B (**3**), demethyltorosaflavone D (**4**), and chrysoeriol-7-*O*-rhamnoside (**5**).

HPLC analysis of AP revealed the presence of kaempferol-*O*-glucoside (**1**), quercetin (**2**), alternanthin B (**3**), demethyltorosaflavone D (**4**), and chrysoeriol-7-*O*-rhamnoside (**5**) (Figure 1). The retention times of the eluted peaks were compared with those of the standard compounds.

The HPLC chromatograms of all standards exhibited acceptable peak separation (Figure S1 and Table S1), and the analytical method was validated for selectivity by the disappearance of undesired peaks in the HPLC chromatograms. The within-day (n = 3) and between-days (n = 6) precision of three concentration levels of standards was found to be precise, as the Relative Standard Deviation (RSD) values were less than 3%. The method accuracy was determined by analytical recovery experiments using standards spiked into the extract at three concentrations [28]. The overall recovery percentages were in the range of 90–110%. Linearity, which was obtained by calculating the regression line using the coefficient of determination (r^2^) of the standard curve of each reference compound, was shown to be good (correlation coefficient r^2^ > 0.99). The limits of detection (LODs) were found to be 0.2, 0.5, 0.5, 2.0, and 0.6 µg/mL for **1**, **2**, **3**, **4**, and **5**, respectively. The limits of quantitation (LOQs) for **1**, **2**, **3**, **4**, and **5** were found to be 0.6, 1, 1, 7, 2 µg/mL, respectively. The results from the validation method of AP were reliable and could be applied for analysis of the five compounds (Table S1). The amounts of the five major constituents present in AP were 1.41 ± 0.26 mg/g extract for **1**, 2.30 ± 0.42 mg/g extract for **2**, 4.36 ± 0.45 mg/g extract for **3**, 31.82 ± 0.76 mg/g extract for **4**, and 2.34 ± 0.42 mg/g extract for **5**.

### 2.4. Effect of AP on D-Galactose-Induced Memory Deficits in Behavioral Tests

**Figure 2:**
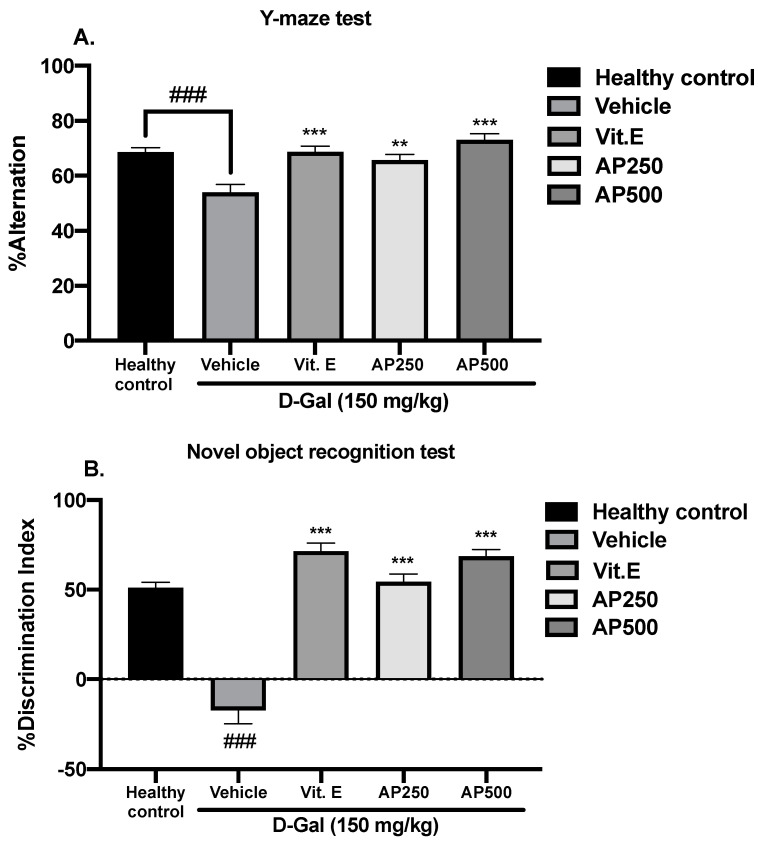
The effect of AP on spatial working memory performance using the Y-maze test (**A**) and the NORT (**B**). Each column represents the mean ± SEM (n = 10). and ### *p* < 0.001 vs. the healthy control group and ** *p* < 0.01, and *** *p* < 0.001 vs. the vehicle-treated group (post hoc Tukey test).

Several behavioral models were used to evaluate D-Gal-induced cognitive dysfunction in the mice. Spatial working memory performance was assessed using the Y-maze task and the novel object recognition test (NORT). The percentage of the spontaneous alternation of the Y-maze test is shown in Figure 2A. The D-Gal vehicle-treated group showed significantly deteriorated cognitive abilities as a reduced percentage of alternation compared with the healthy control group (*p* < 0.001). Treatment with vitamin E (100 µg/kg/day) and AP (250 and 500 mg/kg/day) restored the percentage of spontaneous alternation in D-Gal-treated mice to the level seen in the healthy control group. For the NORT (Figure 2B), the healthy control group spent significantly more time exploring the novel object than the familiar one, while the vehicle-treated mice failed to discriminate between these two objects, indicating that D-Gal impaired object recognition. On the other hand, the D-Gal-treated mice showed a significantly improved discrimination performance in the test phase after administration of vitamin E (100 µg/kg/day) and AP (250 and 500 mg/kg/day) for 8 weeks (for detailed statistical analysis, see Tables S2 and S3). 

**Figure 3:**
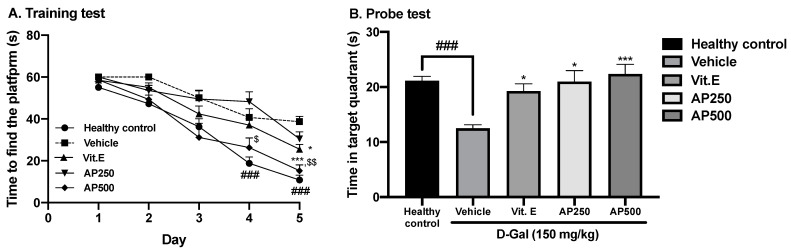
Latency time of training the mice to find the target platform (n = 10). Each data point represents the mean ± SEM of latency, four trials/day for five days (**A**). The effect of AP on the D-Gal-induced cognitive deficit in the MWMT (**B**). Each column represents the mean ± SEM (n = 10). ### *p* < 0.001 vs. the healthy control group; * *p* < 0.05 and *** *p* < 0.001 vs. the vehicle-treated group; and ^$^ *p* < 0.05 and ^$$^ *p* < 0.01 vs. AP (post hoc Tukey test).

Working memory was evaluated by the Morris water maze test (MWMT). Figure 3A shows that the time needed to find the submerged platform in the MWMT progressively decreased over the five-day training period for all groups. However, on day 5, the D-Gal-treated mice spent significantly less time in the target quadrant than the healthy control mice, indicating that D-Gal induced learning and memory deficits (Figure 3B). Oral administration of either vitamin E (100 mg/kg/day) or AP (250 and 500 mg/kg/day) to D-Gal-induced mice significantly increased the time spent in the target quadrant compared to the vehicle-treated mice, indicating an improvement in working memory function (for detailed statistical analysis, see Tables S4.1 and S4.2).

### 2.5. AP Restored Superoxide Dismutase (SOD) and Catalase (CAT) Activities in the Hippocampus and Frontal Cortex

**Figure 4:**
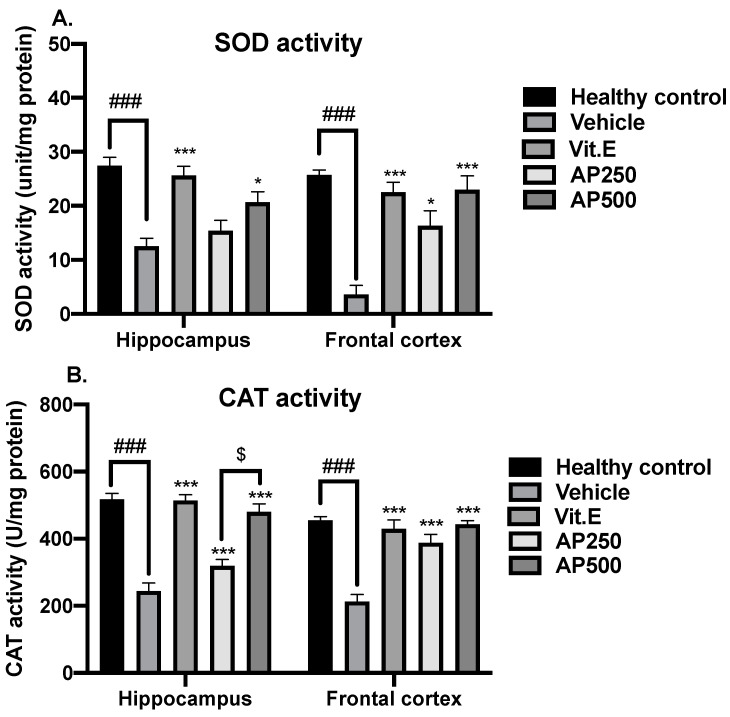
The effect of AP on antioxidant enzymes in the hippocampus and frontal cortex (n = 6). Each data point represents the mean ± SEM. The SOD and CAT activities are presented in panels (**A**,**B**), respectively. ### *p* < 0.001 vs. the healthy control group; * *p* < 0.05 and *** *p* < 0.001 vs. the vehicle-treated group; and ^$^ *p* < 0.05 vs. AP (post hoc Tukey test).

D-Gal induced oxidative stress through a significant decrease in the activities of the antioxidant enzymes SOD and CAT in both the hippocampus and frontal cortex compared to the healthy control group (Figure 4). Administration of either AP at 250 and 500 mg/kg/day or vitamin E restored the activities of SOD and CAT to healthy control levels in both brain regions (for detailed statistical analysis, see Tables S5.1 and S5.2).

### 2.6. AP Alleviated Neuroinflammation and Delayed Telomere Shortening in the Hippocampus and the Frontal Cortex

**Figure 5:**
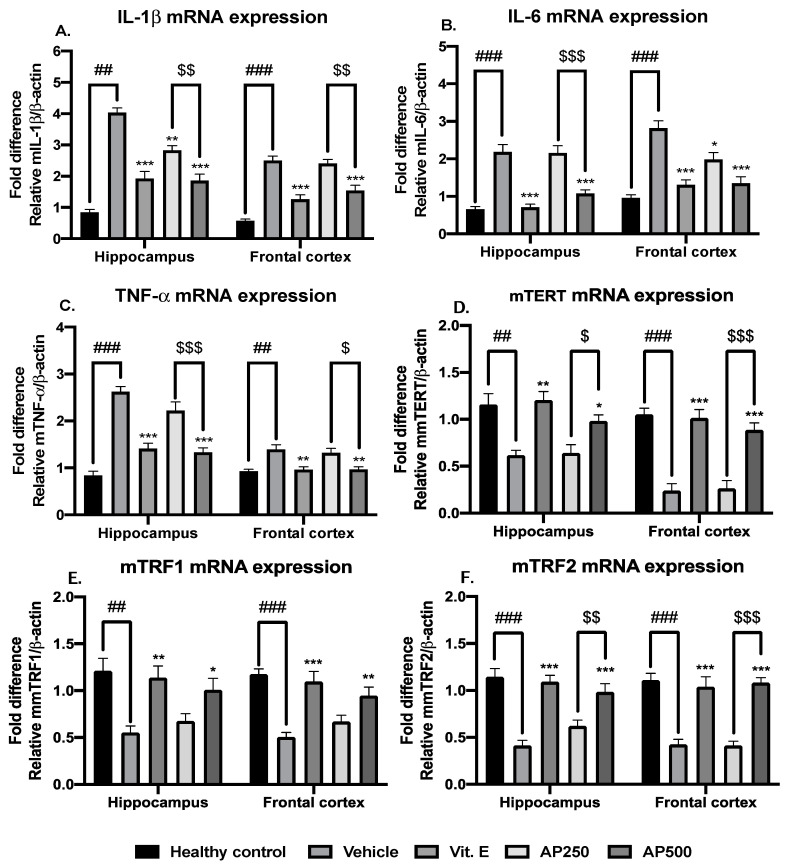
Anti-inflammatory effects and alleviating effects of AP on aging. Each data point represents the mean ± SEM. For the interpretation of the inflammatory pathway, the results are shown in panels (**A**–**C**). The telomere-attrition-relating process is indicated in panels (**D**–**F**). ## *p* < 0.01 and ### *p* < 0.001 vs. the healthy control group; * *p* < 0.05, ** *p* < 0.01, and *** *p* < 0.001 vs. the vehicle-treated group; and ^$^ *p* < 0.05, ^$$^ *p* < 0.01, and ^$$$^ *p* < 0.001 vs. the AP-treated group (post hoc Tukey test).

There was significantly more neuroinflammation and telomere attrition in D-Gal-induced mice compared with the healthy control group. Administration of AP to the D-Gal-induced mice decreased the levels of the proinflammatory cytokines IL-1β, IL-6, and TNF-α and enhanced the levels of the factors associated with telomere elongation and stabilization (mTERT, mTRF1, and mTRF2), which are critical for delaying aging. The results are shown in Figure 5 (for detailed statistical analysis, see Tables S6.1–S6.6).

## 3. Discussion

Since aging induces memory deficits via multiple pathogenic pathways, the classical approach that modulates only one target may not be suitable to tackle aging-induced memory impairment. Thus, searching for compounds that act on multiple targets in the pathologic cascade has been an effective strategy to delay the onset of cognitive symptoms in age-related diseases [5,27,29]. Bearing this paradigm in mind, we designed experiments to evaluate the effect of AP on memory deficits in D-Gal-induced brain aging in mice by examining its effects on antioxidant enzymes, neuroinflammation, and telomere shortening.

Our previous investigation of AP revealed the presence of several flavones and showed that AP and its flavone constituents inhibited β-amyloid (Aβ) aggregation in OVX mice [26,27]. Moreover, AP also downregulated neuroinflammatory cytokines in the hippocampus and frontal cortex of OVX mice [27]. Therefore, it appears that the flavones present in AP could be responsible for the prevention of both oxidative stress and neuroinflammation [26,27]. For this reason, we hypothesized that AP could alleviate neurodegeneration and delay the progression of aging-induced dementia [30]. Our previous study also revealed that AP exhibited antidementia effects that were mediated via estrogenic activity in the OVX mouse model [27]. Thus, it was of great interest to investigate the effects of AP on age-induced dementia using a D-Gal-induced aging model in male mice. The key findings of this investigation are that D-Gal has a detrimental impact on cognition by triggering telomere shortening while inducing proinflammatory cytokines and decreasing antioxidant enzyme activity inside the hippocampus and frontal cortex. Interestingly, these phenomena can be reversed by treatment with AP.

Although there are several animal models used for investigating senescence and dementia, our results demonstrated that D-Gal administration is an effective approach for mimicking aging, which is supported by previous findings by other groups [21,22,31]. During aging, telomere shortening results in cell cycle arrest and reduced proliferative ability, which contribute to a decline in tissue regeneration [16]. Thus, neurons tend to be damaged without being repaired, leading to neurodegenerative disorders. Our findings reveal that chronic administration of D-Gal (8 weeks) accelerated aging effects through diminished expression of the telomere stabilizing factors mTERT, mTRF1, and mTRF2. Neurons are a critical target of telomere attrition with their limited capacity for repair. Interestingly, oral administration of AP or vitamin E reversed these effects by stimulating telomere elongation and stabilizing neurons by enhancing these essential factors. These results indicate that treatment of D-Gal-induced mice with AP helped to delay the aging process via restored telomere regulation.

In the D-Gal-induced aging model, neuroinflammation occurred after cellular components were damaged by ROS [31,32,33] and the hyperactivation of inflammatory responses led to neurodegeneration. D-Gal induced a significant overproduction of the proinflammatory cytokines IL-1ß, IL-6, and TNF-α in the brain. Administration of AP at a dose of 500 mg/kg/day significantly ameliorated neuroinflammation in a dose-dependent manner. A similar effect was also observed with vitamin E.

Antioxidant activity is one of the critical activities responsible for amelioration of memory deficits. AP was evaluated for its total phenolic and total flavonoid contents and antioxidant activity. The ORAC and FRAP assays indicated free radical scavenging mechanisms via hydrogen atom transfer and single electron transfer, respectively [34], and the antioxidant activity determined in these assays was correlated with the presence of phenolic and flavonoid compounds. AP also upregulated SOD and CAT in the hippocampus and frontal cortex brain regions of mice. Subcutaneous administration of D-Gal to mice induced levels of ROS that were too high to be counteracted by the endogenous antioxidant systems and caused oxidative stress. Consequently, SOD and CAT reached their capacities and their activities declined, which is a main characteristic of aging. This concept was supported by a previous study by Liu et al. [21]. On the other hand, mice orally administered either 250 or 500 mg/kg/day of AP showed significantly recovered antioxidant enzyme activities, similar to those observed with the administration of vitamin E.

Administration of D-Gal to rodents over a long period of time causes cognitive problems such as short- and long-term memory deficits, which are similar to those of Alzheimer’s disease (AD) [21,32,35]. The Y-maze test and NORT were performed to determine short-term memory deficits since they measure the ability of mice to remember and navigate within a physical environment. Mice administered with D-Gal at 150 mg/kg/day for 8 weeks showed significant declines in short-term memory. D-Gal-induced mice could not recognize the surrounding environment in the Y-maze test nor discriminate between two objects in the NORT [31,36]. Mice administered with AP showed improved distinguishable behavior for novel and familiar locations in the Y-maze test and improved object discrimination in the NORT. The MWMT evaluates long-term memory, which is related to working memory, a temporary storage system that facilitates holding and manipulating information for brief periods of time [22,32,37]. This task requires keeping multiple pieces of information in mind simultaneously, such as problem-solving, reasoning, and decision-making, which involve the frontal cortex and hippocampus [38,39]. Administration of D-Gal caused a significant loss of learning and memory as demonstrated by more escape latency time and less time spent in the target quadrant. However, oral administration of AP and vitamin E enhanced learning ability and recognition, since the mice were able to recognize the platform located at a certain position and learn to survive in a dangerous environment. These findings are in agreement with a recent study by Dong and Liu [35] which showed that senescent mice (8 weeks after 150 mg/kg D-Gal injection) exhibited worse information acquisition and memory retention.

HPLC analysis of AP revealed the presence of kaempferol-*O*-glucoside (**1**) and quercetin (**2**). In addition, five compounds were identified as markers for quality control and used to perform the HPLC analytical method validation. Thereafter, all the results obtained from the analytical process at 330 nm met the acceptance criteria due to the physicochemical properties of flavones, which usually absorb in the UV spectrum at 304–350 nm [40]. So, the HPLC method performed in this study is suitable for the quality control of *A. philoxeroides* raw materials.

## 4. Materials and Methods 

### 4.1. Determination of Total Phenolic and Flavonoid Contents

The total phenolic content was determined by the Folin–Ciocalteu assay. The Folin–Ciocalteu reagent (Sigma-Aldrich, St. Louis, MO, USA) was used to determine the phenolic content and gallic acid (Sigma-Aldrich, St. Louis, MO, USA) was used as the standard. The result was reported as mg gallic acid equivalents (GAE) per gram of extract. The total flavonoid content was evaluated using the aluminum chloride colorimetric method and reported as quercetin equivalents (QE) per gram of extract, as previously described by Kandeda et al. [36]. Quercetin (Sigma-Aldrich, St. Louis, MO, USA) was used as the standard.

### 4.2. Determination of Radical Scavenging Capacity

#### 4.2.1. Oxygen Radical Absorbance Capacity (ORAC) Assay

The ORAC assay was performed by fluorometric measurements (Perkin Elmer Inc., Waltham, MA, USA) according to the method described by Čagalj et al. [10] with modifications. The reagents used in this assay were purchased from Sigma-Aldrich (St. Louis, MO, USA). The reaction was measured every 5 min, up to 180 cycles. Three replicates of the results were expressed in μM of Trolox Equivalents (TE).

#### 4.2.2. Ferric Reducing Antioxidant Power (FRAP) Assay

The FRAP assay was performed following the method previously described by Skroza et al. [41] with modifications. The FRAP reagent (Sigma-Aldrich, St. Louis, MO, USA) was freshly prepared as a mixture of reagents. Three replicates of the results were expressed in μM of Fe^2+^.

### 4.3. High-Performance Liquid Chromatography (HPLC) Analysis of AP and the Validation Method

AP was analyzed by a reversed-phase HPLC system using a Hypersil™ ODS column (Agilent Technologies Inc., Santa Clara, CA, USA; 4.6 × 250 mm, 5 µm). Acetonitrile (solvent A) and 10% 2-propanol in 1% formic acid aqueous solution (solvent B) were used as the mobile phases, with gradient elution as follows: 5–10% A (5 min), 10–70% A (30 min), 70–75% A (5 min), 75–80% A (5 min), 80–80% A (10 min), and 80–95% A (5 min). The proportional mixture of mobile phases was controlled by the flow rate of 1 mL/min. The extract was prepared as a stock solution (1 mg/mL) in methanol. Kaempferol-3-*O*-glucoside (**1**), quercetin (**2**), alternanthin B (**3**), demethyltorosaflavone D (**4**), and chrysoeriol-7-*O*-rhamnoside (**5**) were prepared and diluted to 1–100 µg/mL as markers prior to establishing standard curves since they were detected at 254, 275, 330, and 370 nm. The analytical method was validated by the analytical performance characteristics, according to the previously described method [28] and as follows. Specificity was the first critical attribute to ensure that the detected signals presented only the desired peaks without significant noise. Linearity indicated the proportional relationship of signal intensity as a function of concentration within a specific range by the coefficient of determination (r^2^) obtained from a linear regression analysis of standard compounds (kaempferol-3-*O*-glucoside = 1–10 µg/mL, quercetin = 2.5–25 µg/mL, alternanthin B = 2.5–25 µg/mL, demethyltorosaflavone D = 10–100 µg/mL, and chrysoeriol-7-*O*-rhamnoside = 2.5–25 µg/mL; n = 5). In addition, the limits of detection (LODs) and the limits of quantitation (LOQs) were determined. Accuracy was evaluated to determine whether the measured amount was close to the actual amount through the recovery percentage of all standard compounds in six determinations (3 concentrations × 3 injections). Precision was validated for within-day (n = 3) and between-days determinations (n = 6) and is reported as a percentage of the relative standard deviation (RSD). 

### 4.4. Animals

Five-week-old male ICR mice (20–30 g; a total of 50 animals) were purchased from Northeast Laboratory Animal Center (Khon Kaen, Thailand) and housed on bedding in cages with water and food provided ad libitum under a 12 h light and dark cycle with controlled temperature (25 ± 2 °C) and constant humidity (45% ± 2%) in the Laboratory Animal Unit of the Faculty of Pharmaceutical Sciences, Khon Kaen University, Khon Kaen, Thailand. The experimental protocol used in the study followed the Guiding Principle for the Care and Use of Animals (NIH Publications #8-23, revised in 2011) and was approved by the Animal Ethics Committee of Khon Kaen University (approval No. IACUC-KKU-77/65, 3 October 2022).

### 4.5. Drug Administrations

After one week of an acclimatization period, all mice were randomly divided into five groups, viz. the healthy control group (corn oil 150 mg/kg/day p.o.), negative control or vehicle-treated group (D-Gal 150 mg/kg/day sc. + corn oil 150 mg/kg/day, p.o.), positive control or Vit. E group (D-Gal 150 mg/kg/day sc. + Vit. E 100 mg/kg/day p.o.), AP250 group (D-Gal 150 mg/kg/day sc. + AP 250 mg/kg/day p.o.), and AP500 group (D-Gal 150 mg/kg/day sc. + AP 500 mg/kg/day p.o.).

**Figure 6:**
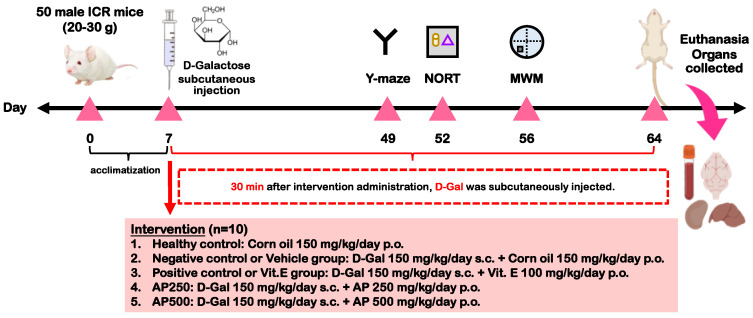
The illustrative diagram of the experimental framework.

The healthy control group was orally administered with corn oil 150 mg/kg/day once daily for 8 weeks. Treatment groups were orally administered with vitamin E (Sigma Aldrich, St. Louis, MO, USA) and AP 30 min prior to subcutaneous administration with D-Gal (Sigma-Aldrich, St. Louis, MO, USA), as illustrated in Figure 6.

### 4.6. Behavioral Assessment

The behavioral tests were performed on week 6. Short-term memory was evaluated by two methods, i.e., the Y-maze test and NORT, according to the method previously described by Khampukdee et al. [27].

The Y-maze apparatus has three V-shaped corridor arms at 60° angles from each other. Mice were allowed to travel through the Y-maze for 8 min after being placed at the end of one arm. When the hind paws of the mouse had completely entered the arm, the entry was considered as complete. Successive entries into the three different arms were interpreted as alternation. Percentage alternation was defined as the ratio of actual to possible alternation (the total number of arm entries minus two), multiplied by 100, according to in the formula below:

% Alternation = [(No. of alternations)/(Total arm entries − 2)] × 100

The NORT was performed in an apparatus consisting of a box with a length of 65 cm, a width of 45 cm, and a height of 45 cm. Two pairs of objects were chosen at random from a set of four objects which differed in shape, surface color, contrast, and texture. During the test session, the positions of the two pairs of objects were shifted. The experiments had two sessions: a sample trial (T1) and a test trial (T2). A habituation test was conducted twenty-four hours prior to experiments. Mice were left individually in the apparatus with no object for 5 min to become used to the box. In T1, mice were placed in front of the wall of the box containing two similar objects which were located symmetrically. Mice were allowed to explore the objects for 5 min. T2 was carried out 30 min after T1. One of the two objects was replaced with a new object (object N). The effect of exploration and the discrimination of the mice was observed for 5 min. Memory was evaluated by assessing the capacity of mice to discern the first object (object F). The time used to explore the familiar object during the 5 min was calculated as the percentage of discrimination index [(N − F)/(N + F)] × 100.

Long-term memory was determined by the Morris water maze task (MWMT), according to the previously described method [27]. The water maze is a circular pool of water (25 ± 1 °C) consisting of four quadrants. In the middle of one quadrant, an escape platform was located 1.0 cm below the water surface in the middle of the pool and was equidistant from the sidewall. The platform for the only escape from the water was situated in the same quadrant in every trial. Three different starting points for mice were placed around the perimeter of the pool. The training period took 5 days to achieve a steady state of escape latency and exclude mice with bad memory. Mice were placed in the pool of water and allowed to swim freely for 60 s, with 4 trials per day. The times at which the mice escaped and reached the platform were documented. On day 6, the platform was taken off the pool and mice were allowed to swim for 60 s per quadrant. The swimming time at the quadrant where the platform used to be was interpreted as a measure of learning ability and memory.

### 4.7. Biochemical Assay

One day after all behavioral tests, the mice were intraperitoneally (i.p.) injected with thiopental sodium (Anesthal^®^, Jagsonpal Pharmaceuticals Ltd., Haryana, India; 60 mg/kg) to anesthetize prior to sacrifice. The hippocampus and frontal cortex were immediately removed. The activities of CAT and SOD and the expression of IL-1ß, IL-6, TNF-α, mTERT, mTRF1, and mTRF2 mRNA were determined in both the hippocampus and frontal cortex. 

#### 4.7.1. Determination of CAT and SOD Activities

A cold phosphate buffer (5 mM, pH 7.4) was used to homogenize the hippocampus and frontal cortex. The activities of SOD and CAT in the homogenate of both brain regions were determined using commercially available kits from Sigma-Aldrich (St. Louis, MO, USA), following the instructions provided by the manufacturer. All the data of SOD and CAT activities were normalized to their total protein concentration in the sample to account for possible differences in protein concentrations between samples. The protein concentrations of the hippocampus and frontal cortex homogenates were determined by the Bradford method, as previously described by Khamphukdee et al. [27].

#### 4.7.2. Quantitative Real-Time Polymerase Chain Reaction (qPCR)

Real-time PCR was used to quantify the expression of IL-1ß, IL-6, TNF-α, mTERT, mTRF1, and mTRF2 mRNA in the frontal cortex and hippocampus. Total RNA was isolated from the tissue using TRIzol^®^ (Thermo Fisher Scientific Inc., San Jose, CA, USA), according to the instructions of the manufacturer. The synthesis of first-strand cDNA was performed utilizing oligo (dT) primers and SuperScript III reverse transcriptase (Thermo Fisher Scientific Inc., located in San Jose, CA, USA). The qPCR was performed using SsoAdvanced™ Universal SYBR Green Supermix (Biorad, Hercules, CA, USA). The primers were synthesized by Macrogen (Seoul, Republic of Korea). Amplification was performed utilizing gene-specific PCR primer sets as follows: (1) -β-actin: 5′-AAC GGT CTC ACG TCA GTG TA-3′ (sense) and 5′-GTG ACA GCA TTG CTT CTG TG-3′ (antisense); (2) -IL-1 ß: 5′- GAC AGC AAA GTG ATA GGC C-3′ (sense) and 5′- CGT CGG CAA TGT ATG TGT TGG -3′ (antisense); (3) -IL-6: 5′-CTT CCA TCC AGT TGC CTT CTT G -3′ (sense) and 5′-AAT TAA-3′ (antisense); (4) -TNF-α: 5′-GCC TCT TCT CAT TCC TGC TTG-3′ (sense) and 5′-CTG ATG AGA GGG AGG CCA TT-3′ (antisense); (5) -mTERT 5′-CTG CGT GTG CGT GCT CTG GAC-3′ (sense) and 5′-CAC CTC AGC AAA CAG CTT GTT CTC-3′ (antisense); (6) -mTRF1 5′-TTC AAC AAC CGA ACA AGT GTC-3′ (sense) and 5′-TCT CTT TCT CTT CCC CCT CC-3′ (antisense); and (7) -mTRF2 5′-GCC CAA AGC ATC CAA AGA C-3′ (sense) and 5′-ACT CCA TCC TTC ACC CAT C-3′ (antisense). After each amplification, a melting curve analysis for each gene was carried out using β-actin mRNA as a control, to which the results were normalized. Relative expressions corresponding to a fold difference were calculated.

### 4.8. Statistical Analysis

The data of the in vitro tests were expressed as the mean ± SD. Behavioral and neurochemical results for each group were presented as mean ± SEM and analyzed by the Student’s *t*-test for the healthy control group and the vehicle-treated group and a one-way ANOVA, followed by a test for multiple comparisons among different groups (the post hoc Tukey test). Differences in *p* values of less than 0.05 were considered statistically significant. SigmaStat^®^ ver. 3.5 (SYSTAT Software Inc., San Jose, CA, USA) was used to analyze the data.

## 5. Conclusions

D-Gal-induced aging in mice is one of the most-used animal models to mimic AD. This model was used to evaluate the effects of AP on short- and long-term memory deficits caused by aging. The results from our study revealed, for the first time, that AP modulated brain aging by delaying the progressive shortening of telomeres through the upregulation of mTERT, mTRF1, and mTRF2 expression. AP also attenuated neuroinflammation via the downregulation of the proinflammatory cytokines IL-1ß, IL-6, and TNF-α while increasing the activities of the antioxidant enzymes SOD and CAT in the hippocampus and frontal cortex, thus effectively improving deficits in both short- and long-term memory in animal behavioral models. The HPLC analysis of AP revealed the presence of several flavones, which appear to be responsible for both the enhancement of antioxidant activity, as well as the restoration of telomere-regulating factor expression, which improved telomere stabilization. An advantage of AP is its use as a food in the northeastern region of Thailand, indicating that it is non-toxic. Therefore, this herb has potential as an anti-aging food supplement.

## Data Availability

Data are contained within the article and supplementary materials.

## Supplementary Materials

The following supporting information can be downloaded at: https://www.mdpi.com/article/10.3390/molecules29020503/s1, Figure S1. HPLC chromatograms of five standards in the ethanol extract of *A. philoxeroides* (**1** = kaempferol-*O*-glucoside, **2** = quercetin, **3** = alternanthin B, **4** = demethyltorosaflavone D, and **5** = chrysoeriol-7-*O*-rhamnoside). Table S1. Validation results of the analytical method for determination of the phytochemical constituents in the ethanol extract of AP. Table S2. Statistical test of the Y-maze test. Table S3. Statistical test of the novel object recognition test (NORT). Table S4.1 Statistical test of the MWMT on the training test. Table S4.2 Statistical test of the MWMT on the probe test. Table S5.1 Statistical test of AP-extract-reversed activity of superoxide dismutase (SOD). Table S5.2 Statistical test of AP-extract-reversed activity of catalase (CAT). Table S6.1 Statistical test of IL-1β mRNA expression in the hippocampus and frontal cortex. Table S6.2 Statistical test of IL-6 mRNA expression in the hippocampus and frontal cortex. Table S6.3 Statistical test of TNF-α mRNA expression in the hippocampus and frontal cortex. Table S6.4 Statistical test of mTERT mRNA expression in the hippocampus and frontal cortex. Table S6.5 Statistical test of mTRF1 mRNA expression in the hippocampus and frontal cortex. Table S6.6 Statistical test of mTRF2 mRNA expression in the hippocampus and frontal cortex.
