# Supplementary material for: Evaluation of the Impact of Alternanthera philoxeroides (Mart.) Griseb. Extract on Memory Impairment in D-Galactose-Induced Brain Aging in Mice through Its Effects on Antioxidant Enzymes, Neuroinflammation, and Telomere Shortening
Source: Molecules. 2024 Jan 19;29(2):503. doi: 10.3390/molecules29020503 (PMC10818586; doi:10.3390/molecules29020503)
Supplement: Supplementary file 1 [file molecules-29-00503-s001.zip › molecules-2727087-supplementary.pdf]

Supplementary Materials

Evaluation of the Impact of *Alternanthera philoxeroides* (Mart.) Griseb. Extract on Memory Deficits in D-Galactose-Induced Brain Aging in Mice Through Effects on Antioxidant Enzymes, Neuroinflammation, and Telomere Shortening

Possatorn Aon-im [1], Orawan Monthakantirat [2], Supawadee Daodee [2], Yaowared Chulikhit [2], Nattapatsorn Sriya [2], Chantana Boonyarat [2], Thanut Chumwangwapee [2], Charinya Khamphukdee [2,*] and Anake Kijjoa [2,3,*]

[1] Graduate School of Pharmaceutical Sciences, Khon Kaen University, Khon Kaen 40002, Thailand; possa.torn@kkumail.com (P.A.)

[2] Faculty of Pharmaceutical Sciences, Khon Kaen University, Khon Kaen 40002, Thailand; oramon@kku.ac.th (O.M.); csupawad@kku.ac.th (S.D.); yaosum@kku.ac.th (Y.C.); nattapatsorn_sriya@kkumail.com (N.S.); chaboo@kku.ac.th (C.B.); thacho@kku.ac.th (T.C.)

[3] ICBAS-Instituo de Ciências Biomédicas Abel Salazar and CIIMAR, Universidade do Porto, Rua de Jorge Viterbo Ferreira 228, 4050-313 Porto, Portugal

* Correspondence: charkh@kku.ac.th (C.K.); ankijjoa@icbas.up.pt (A.K.); Tel.: +351-220428331 (A.K.)

1. Phytochemical Analysis by High-Performance Liquid Chromatography (HPLC) and Validation Method

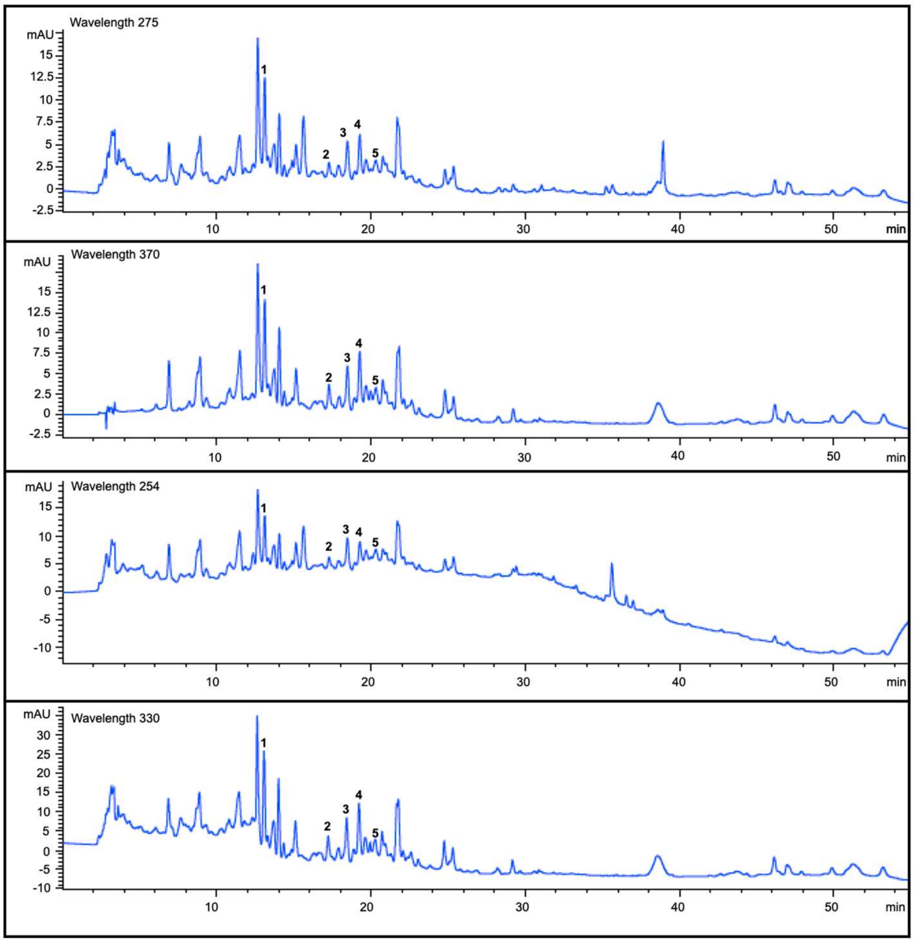

Figure S1. HPLC chromatograms of five standards in the ethanol extract of *A. philoxeroides* (1 = kaempferol-*O*-glucoside, 2 = quercetin, 3 = alternanthin B, 4 = demethyltorosaflavone D, 5 = chrysoeriol-7-*O*-rhamnoside

Table S1. Validation results of the analytical method for determination of phytochemical constituents in the ethanol extract of AP

**Parameter**		**Compounds**				
		**(1)**	**(2)**	**(3)**	**(4)**	**(5)**
Retention Time (min)		13.10	17.27	18.47	19.27	20.32
Linearity	Range (μg/mL)	1-10	2.5-25	2.5-25	10-100	2.5-25
	Coefficient determination (R^2)	0.9993 ± 0.0002	0.9997 ± 0.0000	0.9997 ± 0.0001	0.9998 ± 0.0002	0.9992 ± 0.0007
LOD (μg/mL)		0.2	0.5	0.5	2.0	0.6
LOQ (μg/mL)		0.6	1.0	1.0	7.0	2.9
Precision (%RSD)	Within day	0.350 – 1.290	0.950 – 1.430	0.820 – 1.430	1.400 – 2.000	0.531 – 1.13
	Between day	0.890 – 1.467	1.210 – 1.810	1.520 – 1.910	1.810 – 1.900	1.630 – 1.680
Accuracy (%Recovery)		108.20 ± 0.812	104.27 ± 0.045	101.538 ± 2.776	102.68 ± 2.765	107.030 ± 2.206
		101.31 ± 3.50	99.09 ± 3.890	96.342 ± 3.207	91.969 ± 5.395	104.490 ± 3.481
		97.97 ± 1.321	99.19 ± 1.270	102.049 ± 1.789	103.55 ± 5.234	99.550 ± 4.340

2. Statistical Analysis Effect of AP extract on D-Gal-Induced Memory Deficits in Behavioral Tests Using Y-maze test

Table S2. Statistical test of Y-maze test.

Test for parametric analysis	*P*	
	The Student's t-test	One-way ANOVA
Normality Test	0.864 (Passed)	0.494 (Passed)
Equal Variance Test	0.116 (Passed)	0.573 (Passed)
Group comparison	The Student's t-test	
Healthy control vs. Vehicle group	$P < 0.001$	
	ANOVA followed by Tukey's post hoc test	
	P	F ($DF_{between\ group}$, $DF_{residual}$)
All group	<0.001	$F(3,36)=12.367$
Vehicle group vs. Vit.E group	<0.001	
Vehicle group vs. AP 250 group	0.006	
Vehicle group vs. AP 500 group	<0.001	
AP 250 group vs. AP 500 group	0.130	
Vit.E group vs. AP 250 group	0.783	
Vit.E group vs. AP 500 group	0.565	

3. Statistical Analysis Effect of A. philoxeroides extract (AP) on D-Gal-Induced Memory Deficits in Behavioral Tests Using Novel Object Recognition Test (NORT)

Table S3. Statistical test of novel object recognition test (NORT).

Test for parametric analysis	*P*	
	The Student's t-test	One-way ANOVA
Normality Test	0.053 (Passed)	0.682 (Passed)
Equal Variance Test	0.102 (Passed)	0.329 (Passed)
Group comparison	The Student's t-test	
Healthy control vs. D-Gal + Vehicle group	$P < 0.001$	
	ANOVA followed by Tukey's post hoc test	
	P	F ($DF_{between\ group}$, $DF_{residual}$)
The test phase trial		
All group	<0.001	$F(3,36)=63.094$
Vehicle group vs. Vit.E group	<0.001	
Vehicle group vs. AP 250 group	<0.001	
Vehicle group vs. AP 500 group	<0.001	
AP 250 group vs. AP 500 group	0.236	
Vit.E group vs. AP 250 group	0.113	
Vit.E group vs. AP 500 group	0.979	

4. Statistical Analysis Effect of AP extract on D-Gal-Induced Memory Deficits in Behavioral Tests Using Morris Water Maze Test (MWMT)

Table S4.1 Statistical test of MWMT on the training test

Test for parametric analysis	*P*	
	The Student's t-test	One-way ANOVA
Day 1-3	-	-
Day 4: Normality Test	0.641 (Passed)	0.596 (Passed)
Equal Variance Test	0.549 (Passed)	0.917 (Passed)
Day 5: Normality Test	0.823 (Passed)	0.715 (Passed)
Equal Variance Test	0.059 (Passed)	0.687 (Passed)
Group comparison	The Student's t-test	
Day 4: Healthy control vs. D-Gal + Vehicle group	$P < 0.001$	
Day 5: Healthy control vs. D-Gal + Vehicle group	$P < 0.001$	
	ANOVA followed by Tukey's post hoc test	
	P	F ($DF_{between\ group}$, $DF_{residual}$)
Day 1-3	-	-
Day 4: All group	0.007	F (3,36)=4.740
Vehicle group vs. Vit.E group	0.925	
Vehicle group vs. AP 250 group	0.593	
Vehicle group vs. AP 500 group	0.088	
AP 250 group vs. AP 500 group	0.040	
Vit.E group vs. AP 250 group	0.254	
Vit.E group vs. AP 500 group	0.282	
Day 5: All group	<0.001	F (3,36)=12.425
Vehicle group vs. Vit.E group	0.010	
Vehicle group vs. AP 250 group	0.168	
Vehicle group vs. AP 500 group	<0.001	
AP 250 group vs. AP 500 group	0.003	
Vit.E group vs. AP 250 group	0.600	
Vit.E group vs. AP 500 group	0.062	

Table S4.2 Statistical test of MWMT on the probe test

Test for parametric analysis	*P*	
	The Student's t-test	One-way ANOVA
Normality Test	0.636 (Passed)	0.347 (Passed)
Equal Variance Test	0.392 (Passed)	0.061 (Passed)
Group comparison	The Student's t-test	
Healthy control vs. D-Gal + Vehicle group	*P* < 0.001	
	ANOVA followed by Tukey's post hoc test	
	P	*F* ($DF_{between\ group}$, $DF_{residual}$)
All group	<0.001	$F(3,36)=8.325$
Vehicle group vs. Vit.E group	0.017	
Vehicle group vs. AP 250 group	0.002	
Vehicle group vs. AP 500 group	<0.001	
AP 250 group vs. AP 500 group	0.911	
Vit.E group vs. AP 250 group	0.851	
Vit.E group vs. AP 500 group	0.468	

5. Statistical Analysis AP extract Reversed Activity of Superoxide Dismutase (SOD) and Catalase (CAT) in the Hippocampus and Frontal cortex in the Frontal cortex and Hippocampus

Table S5.1 Statistical test of AP extract Reversed Activity of Superoxide Dismutase (SOD)

Test for parametric analysis	*P*	
	The Student's t-test	One-way ANOVA
Hippocampus: Normality Test	0.689 (Passed)	0.158 (Passed)
Equal Variance Test	0.956 (Passed)	0.969 (Passed)
Frontal cortex: Normality Test	0.468 (Passed)	0.178 (Passed)
Equal Variance Test	0.149 (Passed)	0.866 (Passed)
Group comparison	The Student's t-test	
Hippocampus: Healthy control vs. D-Gal + Vehicle group	*P* < 0.001	
Frontal cortex: Healthy control vs. D-Gal + Vehicle group	*P* < 0.001	
	ANOVA followed by Tukey's post hoc test	
	P	*F* ($DF_{between\ group}$, $DF_{residual}$)
Hippocampus		
All group	0.004	$F(3,20)=11.126$
Vehicle group vs. Vit.E group	<0.001	
Vehicle group vs. AP 250 group	0.657	
Vehicle group vs. AP 500 group	0.017	
AP 250 group vs. AP 500 group	0.170	
Vit.E group vs. AP 250 group	0.003	
Vit.E group vs. AP 500 group	0.218	

Frontal cortex		
All group	0.012	F(3,20)=15.773
Vehicle group vs. Vit.E group	<0.001	
Vehicle group vs. AP 250 group	0.004	
Vehicle group vs. AP 500 group	<0.001	
AP 250 group vs. AP 500 group	0.291	
Vit.E group vs. AP 250 group	0.246	
Vit.E group vs. AP 500 group	0.999	

Table S5.2 Statistical test of AP Reversed Activity of Catalase (CAT)

Test for parametric analysis	P	
	The Student's t-test	One-way ANOVA
Hippocampus: Normality Test	0.165 (Passed)	0.200 (Passed)
Equal Variance Test	0.882 (Passed)	0.877 (Passed)
Frontal cortex: Normality Test	0.119 (Passed)	0.297 (Passed)
Equal Variance Test	0.330 (Passed)	0.657 (Passed)
Group comparison	The Student's t-test	
Hippocampus: Healthy control vs. D-Gal + Vehicle group	$P < 0.001$	
Frontal cortex: Healthy control vs. D-Gal + Vehicle group	$P < 0.001$	
	ANOVA followed by Tukey's post hoc test	
	P	F ($DF_{between\ group}$, $DF_{residual}$)
Hippocampus		
All group	<0.001	F(3,20)=34.005
Vehicle group vs. Vit.E group	<0.001	
Vehicle group vs. AP 250 group	<0.001	
Vehicle group vs. AP 500 group	<0.001	
AP 250 group vs. AP 500 group	0.016	
Vit.E group vs. AP 250 group	<0.05	
Vit.E group vs. AP 500 group	0.647	
Frontal cortex		
All group	0.004	F(3,20)=24.828
Vehicle group vs. Vit.E group	<0.001	
Vehicle group vs. AP 250 group	<0.001	
Vehicle group vs. AP 500 group	<0.001	
AP 250 group vs. AP 500 group	0.291	
Vit.E group vs. AP 250 group	0.532	
Vit.E group vs. AP 500 group	0.968	

6. Statistical Analysis of AP Extract Alleviated Neuroinflammation as well as Delayed Telomere Shortening in the Hippocampus and the Frontal Cortex

Table S6.1 Statistical test of IL-1β mRNA expression in hippocampus and frontal cortex

Test for parametric analysis	P

	The Student's t-test	One-way ANOVA
Hippocampus: Normality Test	0.411 (Passed)	0.535 (Passed)
Equal Variance Test	0.331 (Passed)	0.701 (Passed)
Frontal cortex: Normality Test	0.458 (Passed)	0.325 (Passed)
Equal Variance Test	0.149 (Passed)	0.551 (Passed)
Group comparison	The Student's t-test	
Hippocampus: Healthy control vs. D-Gal + Vehicle group	$P < 0.001$	
Frontal cortex: Healthy control vs. D-Gal + Vehicle group	$P < 0.001$	
	ANOVA followed by Tukey's post hoc test	
	P	F ($DF_{between\ group}$, $DF_{residual}$)
Hippocampus		
All group	<0.001	$F(3,20)=29.79$
Vehicle group vs. Vit.E group	<0.001	
Vehicle group vs. AP 250 group	0.001	
Vehicle group vs. AP 500 group	<0.001	
AP 250 group vs. AP 500 group	0.007	
Vit.E group vs. AP 250 group	0.013	
Vit.E group vs. AP 500 group	0.993	
Frontal cortex		
All group	<0.001	$F(3,20)=18.332$
Vehicle group vs. Vit.E group	<0.001	
Vehicle group vs. AP 250 group	0.965	
Vehicle group vs. AP 500 group	<0.001	
AP 250 group vs. AP 500 group	0.002	
Vit.E group vs. AP 250 group	<0.001	
Vit.E group vs. AP 500 group	0.525	

Table S6.2 Statistical test of IL-6 mRNA expression in hippocampus and frontal cortex

Test for parametric analysis	P	
	The Student's t-test	One-way ANOVA
Hippocampus: Normality Test	0.468 (Passed)	0.768 (Passed)
Equal Variance Test	0.076 (Passed)	0.054 (Passed)
Frontal cortex: Normality Test	0.657 (Passed)	0.261 (Passed)
Equal Variance Test	0.126 (Passed)	0.901 (Passed)
Group comparison	The Student's t-test	
Hippocampus: Healthy control vs. D-Gal + Vehicle group	$P < 0.001$	
Frontal cortex: Healthy control vs. D-Gal + Vehicle group	$P < 0.001$	
	ANOVA followed by Tukey's post hoc test	
	P	F ($DF_{between\ group}$, $DF_{residual}$)
Hippocampus		
All group	<0.001	$F(3,20)=25.337$

Vehicle group vs. Vit.E group	<0.001	
Vehicle group vs. AP 250 group	0.999	
Vehicle group vs. AP 500 group	<0.001	
AP 250 group vs. AP 500 group	<0.001	
Vit.E group vs. AP 250 group	<0.001	
Vit.E group vs. AP 500 group	0.340	
Frontal cortex		
All group	<0.001	$F(3,20)=22.017$
Vehicle group vs. Vit.E group	<0.001	
Vehicle group vs. AP 250 group	0.014	
Vehicle group vs. AP 500 group	<0.001	
AP 250 group vs. AP 500 group	0.074	
Vit.E group vs. AP 250 group	0.053	
Vit.E group vs. AP 500 group	0.998	

Table S6.3 Statistical test of TNF-α mRNA expression in hippocampus and frontal cortex

Test for parametric analysis	*P*	
	The Student's t-test	One-way ANOVA
Hippocampus: Normality Test	0.141 (Passed)	0.373 (Passed)
Equal Variance Test	0.600 (Passed)	0.116 (Passed)
Frontal cortex: Normality Test	0.449 (Passed)	0.368 (Passed)
Equal Variance Test	0.185 (Passed)	0.812 (Passed)
Group comparison	The Student's t-test	
Hippocampus: Healthy control vs. D-Gal + Vehicle group	$P < 0.001$	
Frontal cortex: Healthy control vs. D-Gal + Vehicle group	$P = 0.007$	
	ANOVA followed by Tukey's post hoc test	
	P	F ($DF_{between\ group}$, $DF_{residual}$)
Hippocampus		
All group	<0.001	$F(3,20)=24.101$
Vehicle group vs. Vit.E group	<0.001	
Vehicle group vs. AP 250 group	0.151	
Vehicle group vs. AP 500 group	<0.001	
AP 250 group vs. AP 500 group	<0.001	
Vit.E group vs. AP 250 group	0.001	
Vit.E group vs. AP 500 group	0.973	
Frontal cortex		
All group	<0.001	$F(3,20)=8.551$
Vehicle group vs. Vit.E group	0.004	
Vehicle group vs. AP 250 group	0.899	
Vehicle group vs. AP 500 group	0.005	
AP 250 group vs. AP 500 group	0.023	
Vit.E group vs. AP 250 group	0.020	
Vit.E group vs. AP 500 group	1.000	

Table S6.4 Statistical test of mTERT mRNA expression in hippocampus and frontal cortex

Test for parametric analysis	*P*	
	The Student's t-test	One-way ANOVA
Hippocampus: Normality Test	0.342 (Passed)	0.764 (Passed)
Equal Variance Test	0.373 (Passed)	0.592 (Passed)
Frontal cortex: Normality Test	0.296 (Passed)	0.192 (Passed)
Equal Variance Test	0.970 (Passed)	0.961 (Passed)
Group comparison	The Student's t-test	
Hippocampus: Healthy control vs. D-Gal + Vehicle group	$P = 0.002$	
Frontal cortex: Healthy control vs. D-Gal + Vehicle group	$P < 0.001$	
	ANOVA followed by Tukey's post hoc test	
	P	F ($DF_{between\ group}$, $DF_{residual}$)
Hippocampus		
All group	<0.001	$F(3,20)=13.731$
Vehicle group vs. Vit.E group	<0.001	
Vehicle group vs. AP 250 group	0.997	
Vehicle group vs. AP 500 group	0.015	
AP 250 group vs. AP 500 group	0.024	
Vit.E group vs. AP 250 group	<0.001	
Vit.E group vs. AP 500 group	0.200	
Frontal cortex		
All group	<0.001	$F(3,20)=24.592$
Vehicle group vs. Vit.E group	<0.001	
Vehicle group vs. AP 250 group	0.997	
Vehicle group vs. AP 500 group	<0.001	
AP 250 group vs. AP 500 group	<0.001	
Vit.E group vs. AP 250 group	<0.001	
Vit.E group vs. AP 500 group	0.688	

Table S6.5 Statistical test of mTRF1 mRNA expression in hippocampus and frontal cortex

Test for parametric analysis	*P*	
	The Student's t-test	One-way ANOVA
Hippocampus: Normality Test	0.691 (Passed)	0.844 (Passed)
Equal Variance Test	0.451 (Passed)	0.395 (Passed)
Frontal cortex: Normality Test	0.515 (Passed)	0.619 (Passed)
Equal Variance Test	0.848 (Passed)	0.379 (Passed)
Group comparison	The Student's t-test	
Hippocampus: Healthy control vs. D-Gal + Vehicle group	$P = 0.001$	
Frontal cortex: Healthy control vs. D-Gal + Vehicle group	$P < 0.001$	

	ANOVA followed by Tukey's post hoc test	
	P	*F* ($DF_{between\ group}$, $DF_{residual}$)
Hippocampus		
All group	0.002	*F*(3,20)=7.209
Vehicle group vs. Vit.E group	0.003	
Vehicle group vs. AP 250 group	0.828	
Vehicle group vs. AP 500 group	0.024	
AP 250 group vs. AP 500 group	0.134	
Vit.E group vs. AP 250 group	0.022	
Vit.E group vs. AP 500 group	0.803	
Frontal cortex		
All group	<0.001	*F*(3,20)=10.558
Vehicle group vs. Vit.E group	<0.001	
Vehicle group vs. AP 250 group	0.505	
Vehicle group vs. AP 500 group	0.006	
AP 250 group vs. AP 500 group	0.112	
Vit.E group vs. AP 250 group	0.008	
Vit.E group vs. AP 500 group	0.578	

Table S6.6 Statistical test of mTRF2 mRNA expression in hippocampus and frontal cortex

Test for parametric analysis	*P*	
	The Student's t-test	One-way ANOVA
Hippocampus: Normality Test	0.499 (Passed)	0.079 (Passed)
Equal Variance Test	0.311 (Passed)	0.882 (Passed)
Frontal cortex: Normality Test	0.208 (Passed)	0.128 (Passed)
Equal Variance Test	0.696 (Passed)	0.059 (Passed)
Group comparison	The Student's t-test	
Hippocampus: Healthy control vs. D-Gal + Vehicle group	*P* < 0.001	
Frontal cortex: Healthy control vs. D-Gal + Vehicle group	*P* < 0.001	

	ANOVA followed by Tukey's post hoc test	
	P	*F* ($DF_{between\ group}$, $DF_{residual}$)
Hippocampus		
All group	<0.001	*F*(3,20)=20.121
Vehicle group vs. Vit.E group	<0.001	
Vehicle group vs. AP 250 group	0.188	
Vehicle group vs. AP 500 group	<0.001	
AP 250 group vs. AP 500 group	0.008	
Vit.E group vs. AP 250 group	<0.001	
Vit.E group vs. AP 500 group	0.697	
Frontal cortex		
All group	<0.001	*F*(3,20)=28.354
Vehicle group vs. Vit.E group	<0.001	

Vehicle group vs. AP 250 group	0.999	
Vehicle group vs. AP 500 group	<0.001	
AP 250 group vs. AP 500 group	<0.001	
Vit.E group vs. AP 250 group	<0.001	
Vit.E group vs. AP 500 group	0.971	
